# The Small Step Early Intervention Program for Infants at High Risk of Cerebral Palsy: A Single-Subject Research Design Study

**DOI:** 10.3390/jcm13175287

**Published:** 2024-09-06

**Authors:** Ann-Kristin G. Elvrum, Silja Berg Kårstad, Gry Hansen, Ingrid Randby Bjørkøy, Stian Lydersen, Kristine Hermansen Grunewaldt, Ann-Christin Eliasson

**Affiliations:** 1 Department of Neuromedicine and Movement Science, Faculty of Medicine and Health Sciences, Norwegian University of Science and Technology, 7491 Trondheim, Norway; 2 Department of Clinical and Molecular Medicine, Faculty of Medicine and Health Sciences, Norwegian University of Science and Technology, 7491 Trondheim, Norway; kristine.grunewaldt@ntnu.no; 3 Clinic of Rehabilitation, St. Olav’s Hospital, Trondheim University Hospital, 7006 Trondheim, Norway; 4 Regional Centre for Child and Youth Mental Health and Child Welfare (RKBU Central Norway), Department of Mental Health, Faculty of Medicine and Health Sciences, Norwegian University of Science and Technology, 7491 Trondheim, Norway; silja.b.karstad@ntnu.no (S.B.K.); stian.lydersen@ntnu.no (S.L.); 5 Child and Adolescent Mental Health Services, St. Olav’s Hospital, Trondheim University Hospital, 7006 Trondheim, Norway; 6 Children and Youth Clinic, St. Olav’s Hospital, Trondheim University Hospital, 7006 Trondheim, Norway; gry.hansen@stolav.no (G.H.); ingrid.randby.bjorkoy@stolav.no (I.R.B.); 7 Neuropediatric Unit, Department of Women’s and Children’s Health, Karolinska Institutet, 17671 Stockholm, Sweden; ann-christin.eliasson@ki.se; 8 Neuropediatric Research Unit, Astrid Lindgren Children’s Hospital, 17176 Stockholm, Sweden

**Keywords:** early intervention, high risk, cerebral palsy, family centered, interprofessional

## Abstract

**Background/Objectives**: Early interventions for infants at high risk of cerebral palsy (CP) are recommended, but limited evidence exists. Our objective was, therefore, to evaluate the effects of the family-centered and interprofessional Small Step early intervention program on motor development in infants at high risk of CP (ClinicalTrials.gov: NCT03264339). **Methods**: A single-subject research design was employed to investigate participant characteristics (motor dysfunction severity measured using the Hammersmith Infant Neurological Examination (HINE) and Alberta Infant Motor Scale (AIMS) at three months of corrected age (3mCA) related to intervention response. The repeated measures Peabody Developmental Motor Scales-2 fine and gross motor composite (PDMS2-FMC and -GMC) and Hand Assessment for Infants (HAI) were analyzed visually by cumulative line graphs, while the Gross Motor Function Measure-66 (GMFM-66) was plotted against reference percentiles for various Gross Motor Function Classification System (GMFCS) levels. **Results**: All infants (*n* = 12) received the Small Step program, and eight completed all five training steps. At two years of corrected age (2yCA), nine children were diagnosed with CP. The children with the lowest HINE < 25 and/or AIMS ≤ 6 at 3mCA (*n* = 4) showed minor improvements during the program and were classified at GMFCS V 2yCA. Children with HINE = 25–40 (*n* = 5) improved their fine motor skills during the program, and four children had larger GMFM-66 improvements than expected according to the reference curves but that did not always happen during the mobility training steps. Three children with HINE = 41–50 and AIMS > 7 showed the largest improvements and were not diagnosed with CP 2yCA. **Conclusions**: Our results indicate that the Small Step program contributed to the children’s motor development, with better results for those with an initial higher HINE (>25). The specificity of training could not be confirmed.

## 1. Introduction

Early interventions in infants with or at high risk of cerebral palsy (CP) aim to take advantage of the activity-dependent neuroplasticity and rapid development that happens during the first years of life [1]. It is emphasized that the heterogeneity of the CP diagnosis requires interventions adapted to the type, topography, and severity of the disorder [2]. However, there is still limited evidence regarding which intervention modes are best suited to promote motor development in infants at various functional levels [3].

Regardless of heterogeneity, a family-centered interdisciplinary approach is recommended, in which the therapists respect and accept the uniqueness of each family, acknowledge the parents as the experts on their child’s needs and abilities, and recognize the importance of a supportive family to promote optimal child development and functioning [4,5]. Interventions should be goal-oriented and at the appropriate level of challenge for the infant, aligning with the latest research on early learning-induced brain plasticity and motor learning principles [1,2,6]. Furthermore, the importance of the infant’s active involvement and frequent practice of targeted activities in a motivating enriched home environment to harness plasticity and learning is highlighted [1,2,7].

In the Small Step program, the above principles are implemented [8]. The intervention is provided daily at home by parents, with coaching once a week by an interprofessional team of healthcare providers responsible for three distinct foci: mobility, hand use, and communication [8]. The efficacy of the Small Step program was investigated through a randomized controlled trial indicating no difference in motor development between the children who received the Small Step Program and those who received standard care [9]. However, secondary analyses indicated that the Small Step program helped the most affected children to catch up by the end of the intervention [9]. This is in contrast to other studies describing that infants with milder brain injury respond better to early intervention than those with severe injury [10]. Furthermore, the specificity of training outcomes could only be suggested for gross motor function, not hand function [11]. Thus, the evidence for the multifaceted Small Step program seems promising, but the results remain inconclusive.

In heterogeneous populations, variability in the magnitude and direction of response to treatment may mask results if randomized controlled trials are used [12]. Single-subject research designs (SSRDs) may, therefore, be advantageous in measuring and describing individual responses to intervention [13]. In SSRDs, multiple baseline designs are used, with each participant serving as his/her own control. This design allows the investigation of characteristics by the participants that may be associated with differential responses to intervention, and if the effects are replicated across individuals, the results can provide powerful evidence supporting or failing to support treatment efficacy [14,15].

The overall aim of this study was, therefore, to evaluate the efficacy of the Small Step early intervention program on motor development in infants at high risk of CP. The main hypothesis was that children would respond differently to the intervention depending on their initial severity of motor dysfunction (i.e., delayed motor development and presence of neurological symptoms). The second hypothesis was that children would show more rapid development within the focus of each specific training step compared to untrained steps.

## 2. Materials and Methods

**Figure 1:**
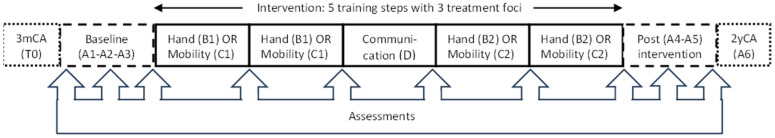
Timeline for the Small Step single-subject research design study. Eligible children were recruited at the regular hospital screening (T0) at three months of corrected age (3mCA). Data collection included three assessments in the baseline phase (A1–A3), one assessment after each training step, and additional two assessments in the post-intervention phase (A4–A5). In addition, one assessment was performed at follow-up (A6) when the children were two years of corrected age (2yCA). The order of the hand function (B) and mobility (C) steps were randomized, while communication (D) was fixed as the third training step.

This SSRD study was performed at St. Olavs Hospital, Trondheim, Norway, from September 2017 to July 2021 (ClinicalTrials.gov: NCT03264339), with each participant serving as his or her own control through multiple testing at baseline and during intervention and withdrawal periods [13]. In the study, we had a baseline and post-intervention phase with repeated measures and no treatment (A). In the intervention period, three alternating treatment foci were implemented: hand function, mobility, and communication (B, C, and D), with the two first foci targeting motor development analyzed in the current paper. The three foci were divided into five different training periods (steps), each supposed to last for six weeks, with approximately one week of testing in between. The hand function and mobility steps were conducted during two time periods, while communication was conducted during one time period. The children were randomized to start with either the hand function or mobility steps, resulting in the following intervention sequence: A-B-C-D-B-C-A or A-C-B-D-C-B-A (see Figure 1). In addition, there was a follow-up assessment when the children were two years of correct age (2yCA). At this age, CP diagnosis was reconsidered.

The study was approved by the Regional Ethical Committee (REC) for Medical Research in Mid-Norway (2016/1366) and was performed in collaboration with the developers of the Small Step program at the Karolinska Institute, Sweden [9,11].

Eligible participants were families of infants with known neonatal risk factors who were diagnosed at high risk of CP at the regular clinical follow-up at three months of corrected age (3mCA). The interim diagnosis was made based on (i) absent or sporadic fidgety movements on the General Movements Assessment (GMA), (ii) abnormal Magnetic Resonance Imaging (MRI), (iii) neurological symptoms (Hammersmith Infant Neurological Examination (HINE) global scores below 57), and (iv) delayed motor development (the Alberta Infant Motor Scale (AIMS) below the 15th percentile) [16,17,18,19,20]. Exclusion criteria were unstable medical conditions, progressive disorders, or diagnosis with a specific syndrome.

### 2.1. Intervention

The Small Step early intervention program is designed for infants from approximately four months of age and is adapted to the child’s development targeting three foci: hand use, mobility, and communication [8]. The intervention is conducted in the children’s home by the parents for 30 min a day, with supervision and coaching once a week by the therapist responsible for each specific training focus. Although different therapists are responsible for each focus area (occupational therapist, physiotherapist, and psychologist, respectively), the overall aim is to strengthen the parent in the role of being the one who knows what is best for their child. The intention is to help parents learn how to use play and the daily environment to promote their child’s development both within the program and outside the training situation. The following general principles are applied: (i) collaborative goal setting with parents to identify short-term and achievable goals; (ii) scaffolding the infant’s self-initiated actions and exploration in meaningful and playful activities; (iii) an enriched home environment with careful toy selection and adaptations to the environment to promote infant activity; and (iv) sufficient intensity and repetition to promote learning [8].

Therapists were introduced to the training principles by the key researchers who developed the Small Step program in Sweden. In addition, they participated in regular team meetings before and during the intervention period. A protocol was developed and used by therapists to promote adherence. We aimed for six coaching sessions during each training step, with possible breaks if the child was ill or due to holidays. Thus, the resulting length for each step could vary slightly. Collaborative goals were set for each week. Parents were asked to note training times, observations, and questions they wanted to discuss in a training diary. Daily training time was calculated based on the parents’ notes as an indicator of treatment feasibility. After each training period, the parents were asked to rate meaningfulness and motivation to continue the program on a scale ranging from one to nine where one indicates low meaningfulness or motivation. In addition, parents were invited to share their perceptions of the program through individual in-depth interviews presented in a separate paper [21].

Intervention fidelity was investigated for the children who completed three or more training periods [8]. One of the sessions in each of the three first training steps was randomly selected to be video-recorded. The videos were later analyzed by a medical student (YMP) using a fidelity checklist specifically developed for this project covering four main areas: (i) The training session structure included a review of the previous week (at the beginning of the session) and goalsetting (at the end of the session) as well as the use of a training diary; (ii) The coaching strategies covered the use of open questions, reflective listening, and the strengthening and encouragement of parents; (iii) The motor learning principles included scaffolding the infant’s self-initiated actions by focusing on motivational toys and activities, providing opportunities for repetition, and exploring the right challenge to promote learning; and (iv) whether the infant’s goals were connected to the intended focus area (hand use, mobility, and communication).

### 2.2. Outcome Measures

To evaluate motor development, we used the Peabody Developmental Motor Scales 2nd edition (PDMS-2), Gross Motor Function Measure-66 (GMFM-66), and Hand Assessment for Infants (HAI) [22,23,24,25]. The assessments were undertaken either in the family’s home or at the hospital by therapists not involved in the delivery of the Small Step intervention (MB and AKGE). The HAI was videotaped and later scored by blinded assessors (BMZ and ES). At two years of CA, we classified the severity of motor function for children with a confirmed CP diagnosis using the Mini-Manual Ability Classification System (Mini-MACS) and the Gross Motor Function Classification System (GMFCS) [26,27].

The PDMS-2 is a norm-referenced measure used to evaluate fine and gross motor skills in children from birth to 6 years of age [23]. It has been found to be responsive to change in toddlers with CP utilizing raw scores [28]. The standard error of measurement (SEM) for the fine and gross motor composites (FMC and GMC, respectively) has been reported to be 3 raw scores, with a higher criterion of 5.88 (i.e., SEM of 1.96 × 3) indicating a change beyond the measurement error [28]. The FMC included the subtests of grasping and visual motor integration, while in the current study, only the stationary and locomotion subtests were included in the GMC. We considered the reflex subtest to be of less relevance, and the object manipulation subtest does not have items for children below 12 months of age [23].

The Hand Assessment for Infants (HAI) is a standardized observation-based assessment for infants 3 to 12 months of age who are at risk of developing CP [22]. It assesses the degree and quality of goal-directed actions performed with each hand separately and with both hands together based on a 10–15 min semi-structured video-recorded play session using age-appropriate toys. Altogether 17 items (12 unimanual and 5 bimanual) are scored on a 3-point rating scale, resulting in a possible HAI total raw score ranging from 0 to 58 points, with a higher score indicating better performance [22,25]. The HAI has been validated by means of Rasch analysis for infants at risk of unilateral CP, and an interval-level scale of 0 to 100 HAI units is used to describe overall hand function in this group of children [22]. In addition, norm-referenced values for the HAI have been established for the raw score based on both hands sum score (HAI total raw score) [29]. Furthermore, there is ongoing work validating the HAI for infants with bilateral involvement. The preliminary results indicate that the HAI works well for infants with bilateral involvement, but a separate HAI unit scale will need to be developed (personal communication). Thus, the HAI total raw score was used in the current study.

The Gross Motor Function Measure-66 (GMFM-66) is a criterion-referenced measure validated to evaluate gross motor change over time in children with CP from 5 months of age [24]. The GMFM-66 has been found to valid, reliable, and responsive to change for children with CP although it may have a floor effect in children at low ability levels [24,30,31,32]. Standard error of measurement (SEM) and 95% confidence intervals for GMFM-66 interval level scores are calculated using the Gross Motor Ability Estimator-2 (GMAE-2) computer program [24]. In addition, distinct gross motor developmental curves with corresponding percentiles across ages and GMFCS levels have been developed based on the GMFM-66 [33]. These motor development curves have been validated with population-based data from Norway, including GMFM-66 measurements from the ages of 5 months [34,35].

### 2.3. Analyses

Descriptive statistics are reported for the treatment fidelity and feasibility results and were calculated using IBM^®^ SPSS^®^ Statistics 2. Demographic characteristics of the children and parents are presented on a group level, while risk factors at 3mCA and intervention-related data are presented individually. We used the MRI classification system (MRICS) to classify blind-scored neuroimaging into five main categories: A = maldevelopments; B = predominant white matter injury; C = predominant grey matter injury; D = miscellaneous; and E = normal findings [18]. Visual analysis that graphically displays data by plotting across time points using cumulative line graphs is an important part of SSRD studies [13,36]. For the PDMS-2, the FMC and GMC raw scores were visually displayed and additionally explored in a table reporting the change beyond measurement error. The HAI total raw scores were visually displayed to explore the development of hand function and plotted against norm-referenced growth curves [29]. For the GMFM-66, interval level data were used to compare visually the gross motor development for each child with a confirmed CP diagnosis at 2yCA with the corresponding Norwegian GMFM-66 reference percentiles within each GMFCS level [35]. In addition, within-child GMFM-66 changes above measurement variability were explored by examining the extent of overlap of the 95% confidence intervals. Furthermore, we explored intervention response related to the severity of motor dysfunction at 3mCA by dividing the participants into three groups based on neurological symptoms: Group (1) HINE scores below 25; Group (2) HINE scores between 25 and 40; and Group (3) HINE scores above 40. Intervention response was also explored relative to AIMS scores at 3mCA.

## 3. Results

Parents of 12 of 14 eligible infants agreed to participate in the study and signed informed consent. See Table 1 for the demographic data. The two families who declined did so due to long travels or ongoing child welfare intervention. At 2yCA, nine children were diagnosed with CP, and five of these had additional impairments (Table 1).

Five of the children were randomized to start with hand function (B) and seven started with mobility training (C) (see Table 2). Eight children completed five training periods, while four completed two to four training periods. Reasons for ending the intervention were demanding family circumstances with several hospital visits (*n* = 1), long-term travel (*n* = 2), and participation in another intensive training for one child (ID4).

### 3.1. Intervention Fidelity and Feasibility

Complete training diaries for the three first training steps were collected from seven families. Based on these diaries, we found that parents provided a median of 5.5 daily training sessions per week (range 3 to 7). However, there was a large variation in time spent on training ranging from 15 min to more than two hours per day. On average, the parents rated the Small Step program as highly meaningful (median 8, range 2 to 9). They were also highly motivated to perform the training (median 9, range 5 to 9).

Intervention fidelity results were based on the review of video-recordings from 27 training sessions for nine children who completed three or more training periods. The videos ranged from 23 to 82 min (median 45 min). All four therapists largely adhered to (i) the training session structure. They used the diary at the beginning (*n* = 16 videos (60%)) and end (*n* = 22 videos (85%)) of the sessions, reviewed the previous week’s training (*n* = 20 videos (74%)) and the child’s achievements (*n* = 26 videos (96%)), as well as defined new goals together with the parents for all training sessions except two that were video-recorded on the last week of the training. (ii) Reflective listening was the most used coaching strategy, with a median of 19 observations (range 10 to 36) throughout the 27 video-recordings, followed by strengthening (median = 9, range 3 to 17), encouragement (median = 8, range 1 to 15), and open questions (median = 7, range 2 to 15). (iii) Integration of motor learning principles was observed throughout all training sessions with the use of motivational, interactive play and repetition to promote child activity. Exploration of opportunities adapted to the child’s level of development was observed for all sessions except one and was more often observed during training sessions targeting mobility (median = 9, range 4 to 20) compared with hand function (median 4, range 2 to 10) and communication (median = 2, range 0 to 7) sessions. (iv) The goals were linked to the focus area in question but sometimes covered two focus areas. Furthermore, most of the training time was spent on activities related to the goals for hand function (84%), mobility (82%), and communication (74%). However, for all training sessions, several play activities included functional abilities also related to the other focus areas.

### 3.2. Changes in Fine and Gross Motor Development

#### 3.2.1. PDMS-2 Fine and Gross Motor Development

Several of the children showed improvements above the measurement error for PDMS-2 fine and gross motor abilities during the baseline period. Furthermore, all children who completed three or more training periods (*n* = 10) showed raw score changes above the measurement error in PDMS-2 FMC (range 10 to 64) and GMC (range 6 to 82) after the total intervention period (see Supplementary Table S1, fifth column).

**Figure 2:**
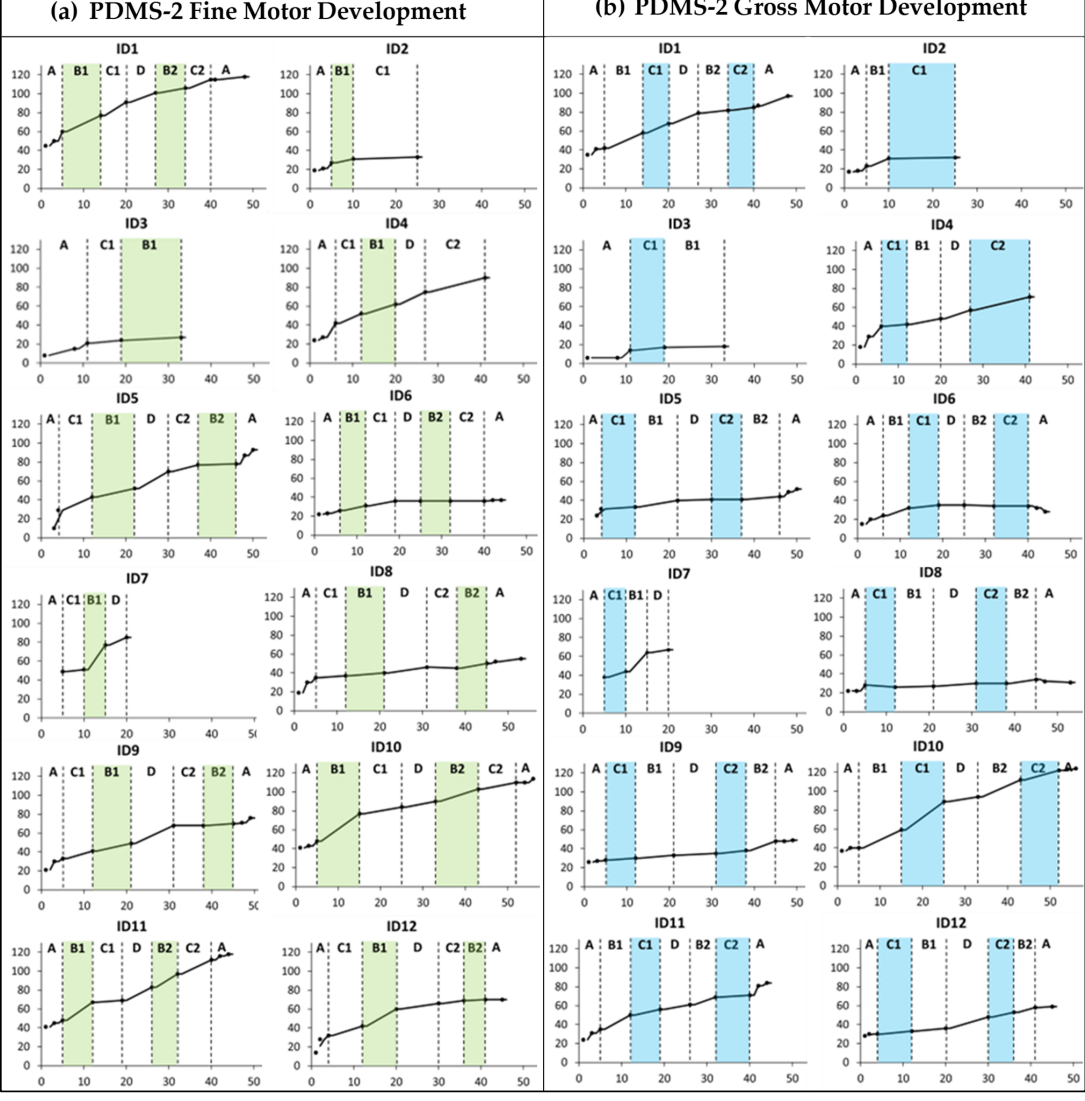
Cumulative slopes for (**a**) Peabody Developmental Motor Scales 2nd edition (PDMS-2) fine motor development and (**b**) PDMS-2 gross motor development measured during the baseline period (A), the training periods targeting hand function (B1 and B2), mobility (C1 and C2), and communication (D), and post-intervention (A). Each participant is represented by an identification (ID) number. The green and blue colors indicate active training periods targeting hand function and mobility, respectively. The *Y*-axis shows PDMS-2 raw scores. The *X*-axis shows number of weeks.

There was no clear trend of improvement related to the specific training foci (Figure 2). However, the cumulative slopes indicate that ID7, ID10, ID11, and ID12 showed more rapid development during the first training period targeting hand function (B1) compared to the baseline and other periods. Furthermore, the cumulative slopes indicate that only ID10 displayed an added gross motor training effect during the first mobility period (C1).

#### 3.2.2. Changes in Bimanual Hand Use Measured with HAI

**Figure 3:**
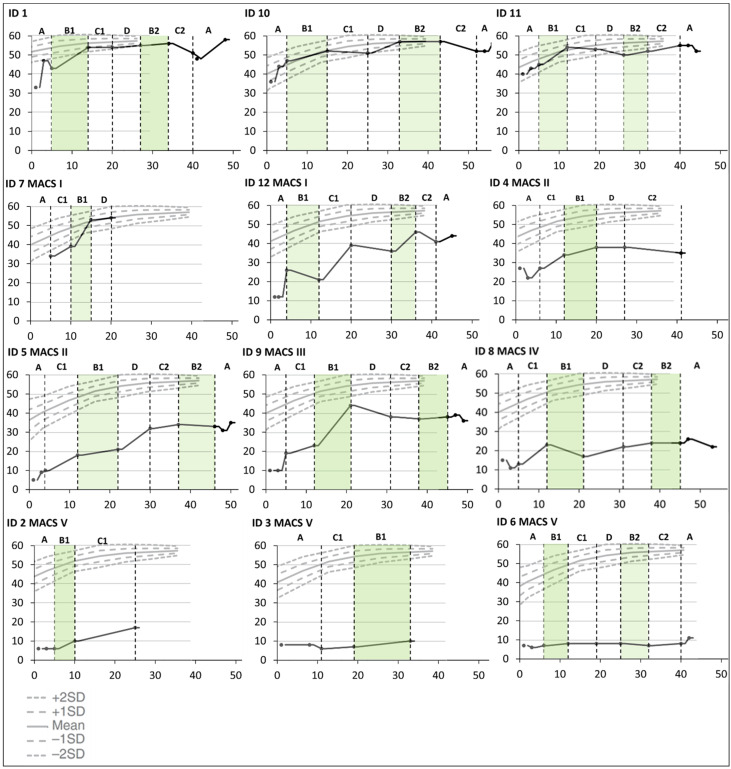
Cumulative slopes for individual Hand Assessment for Infants (HAI) total raw scores are plotted against norm-referenced growth curves for HAI total raw scores [28] during the baseline period (A), the training periods targeting hand function (B1 and B2), mobility (C1 and C2), and communication (D), and post-intervention (A). Each participant is represented by an identification (ID) number. The green color indicates active training periods targeting hand function. The *Y*-axis shows HAI total raw scores. The *X*-axis shows number of weeks after inclusion in the Small Step program.

There were some improvements over time in bimanual hand use for all children except the two who were classified at Mini-MACS level V. This was only related to the hand function period for one child (ID9), and several improved considerably from the first to the last testing in the baseline period (ID1, ID5, ID9, ID10, and ID12). The three children who did not end up with a CP diagnosis (ID1, ID10, and ID11) had a noticeable increase in hand function during the first training period targeting hand function and were thereafter close to the ceiling level (see Figure 3).

#### 3.2.3. GMFM-66 Changes Related to Norwegian GMFM-66 Percentiles

**Figure 4:**
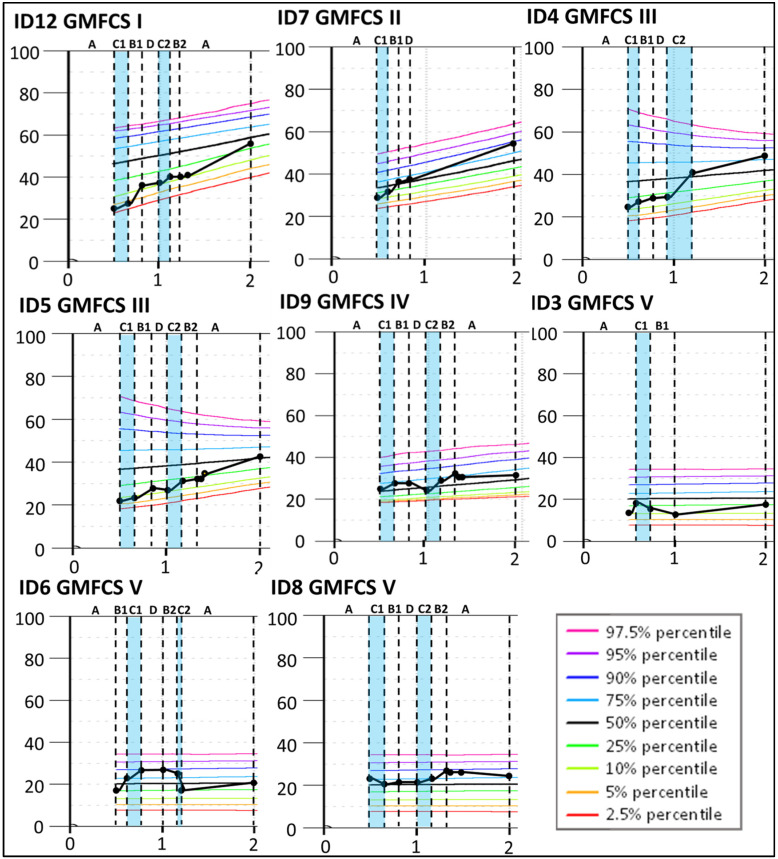
Individual Gross Motor Function Measure-66 (GMFM-66) interval data for the participants with a confirmed CP diagnosis at 2 years of corrected age. The GMFM-66 cumulative slopes are plotted according to the GMFM percentiles for the corresponding GMFCS classification level [35] during the baseline period (A), the training periods targeting hand function (B1 and B2), mobility (C1 and C2), and communication (D), and post-intervention (A). Each participant is represented by an identification (ID) number. The blue color indicates active training periods targeting hand mobility. The *Y*-axis shows GMFM-66 interval level scores. The *X*-axis shows years of age.

The results indicate that four children classified at GMFCS levels I–III (ID12, ID7, ID4, and ID5) improved more than expected according to their initial curve during the Small Step program (see Figure 4) and continued to increase the GMFM-66 score until 2yCA. The largest improvement was indicated for ID4 during the second period targeting mobility (C2) with a change of 11.4, clearly exceeding measurement variability, as indicated by the 95% confidence interval. Similar changes, although smaller, were found for ID12 (change 8.6), ID7 (change 4.7), and ID5 (change 4.4) during the first training period targeting hand function (B1). For children classified at GMFCS levels IV and V, their gross motor development largely corresponded to the expected GMFM-66 curves.

### 3.3. Characteristics of Children Related to Intervention Response

The severity of neurological symptoms measured with the HINE seemed to be associated with differential response to intervention. HINE global scores below 25 (Table 3: Group 1) were indicative of severe CP (GMFCS V) with no or minor fine and gross motor development during the Small Step intervention (see Figure 2, Figure 3 and Figure 4). These children had predominantly white matter injuries or miscellaneous brain development.

For Group 2, with HINE global scores between 25 and 40, four of five children were later classified at GMFCS levels I–III (ID4, ID5, ID7, and ID12). These children improved their GMFM-66 scores above the initial percentiles (Figure 4). All five children showed fine motor improvements over the course of the Small Step program and were later classified at Mini-MACS levels I–III (Figure 2 and Figure 3, Table S1).

For Group 3, with HINE global scores above 40, three of the children (ID1, ID10, and ID11) showed large motor developments (Figure 2 and Figure 3 and Table S1) and did not receive a CP diagnosis at 2yCA. These children had sporadic fidgety movements at 3mCA. One child (ID2) received a diagnosis of CP and was later classified at GMFCS and Mini-MACS level V. This child was classified with maldevelopment of the brain and absent fidgety movements at 3mCA.

In addition, there seems to be an association between very low motor abilities measured with AIMS at 3mCA and a minimal effect of the intervention. The four children with AIMS scorings at or below 6 points (ID2, ID3, ID6, and ID8) had PDMS-2 FMC and GMC total change scores of ≤15 after the Small Step intervention and were later classified at GMFCS level V or Mini-MACS level IV-V (Table 3 and Table S1). For children with a confirmed CP diagnosis and AIMS scores between 7 and 15 at 3mCA (Group 2), there does not seem to be an association with intervention response.

## 4. Discussion

Our results indicate that the Small Step program as a whole contributed to the children’s development, but the specificity of training could not be confirmed. Of the eight children who responded well to the intervention, three did not receive a confirmed CP diagnosis at two years of age, while five with a confirmed CP diagnosis were classified at GMFCS levels I–IV and Mini-MACS levels I–III. Furthermore, the children with the most severe neurological symptoms (HINE < 25) and lowest motor abilities (AIMS ≤ 6) at 3mCA showed only minor motor improvements during the Small Step program and were later classified at GMFCS level V and Mini-MACS levels IV–V.

### 4.1. Treatment Response Related to Initial Severity

The results from our study support the main hypothesis that children would respond differently depending on their initial severity of motor dysfunction. The three children who did not end up with a CP diagnosis had the highest HINE global scores initially and responded well to the intervention. This corresponds with a recent review indicating that for high-risk infants who do not get a CP diagnosis, early developmental interventions may improve motor outcomes in infancy more than usual care [37]. Among the children with a confirmed CP diagnosis, those with HINE global scores between 25 and 40 at 3mCA showed steady fine motor improvements during the Small Step program, and two children were able to walk unaided or by holding on to furniture at two years of age (GMFCS I–II). The latter outcome was better than expected considering that HINE global scores below 40 have been found to be associated with severe CP and inability to sit independently [20].

The children with the most severe motor dysfunction initially displayed limited motor development and had several additional impairments impeding the time windows when they were ready to play. Even so, the parents described how they valued learning to adapt play activities to the child’s ability level to enable participation and engagement in enjoyable activities in daily family life [21]. The Australian GAME (Goals–Activity–Motor Enrichment) study also reported limited intervention response in children with the most severe brain injuries [10], while secondary analyses from the Small Step RCT study indicated that children with the most impaired motor function initially improved more when receiving the early intervention program compared to usual care [9]. However, the numbers were small, emphasizing the need for further research to identify the active ingredients in family-centered early interventions fine-tuned for children at high risk of the most severe CP types, possibly identified early by HINE global scores ≤ 25 [38]. The guidelines recommend that specific rehabilitation programs should focus on building strong parent–infant relationships and promoting child-readiness for play and learning through the prevention of complications related to sleep, nutrition, pain, and musculoskeletal impairments [2]. Furthermore, environmental optimization (i.e., suitable toys, appropriate positioning, adapted seating, and assistive devices) may enable active participation, communication, and learning but needs to be adjusted to each family building on their strengths and preferences [4,38]. This is not significantly different from the recommendations described for children with CP classified at GMFCS and MACS levels I-III, but when less motor development is expected, it may be even more important to explore possibilities together with parents for their child to live a good, fulfilling life now and in the future [4,21].

### 4.2. Specificity of Training

In general, we did not find that children had a more rapid development within the foci of each specific training step when compared to untrained foci. Thus, the specificity of training could not be confirmed, although some children seemed to improve more during the first period targeting hand function compared to the other training steps. The lack of training specificity and possible heightened intervention response during the first training period are supported by previous early intervention studies [11,39]. Several factors may contribute to the lack of training specificity. One possible reason is that parents, once they learn how to engage their child in playful and motivating activities focused on specific treatment goals, may incorporate these activities naturally into daily life. This is supported by our qualitative study, in which parents talked about how they integrated what they learned into everyday play and daily routines [21]. A recent review suggests that it may not be possible to evaluate fully the contribution of separate intervention elements when provided within a comprehensive family-centered intervention program by a coordinated multidisciplinary team of therapists [5]. Rather, evaluating the effects of the total intervention package might be required.

Furthermore, some children in our study improved their motor development considerably during the baseline period suggesting that young infants with injuries in the immature brain may show spontaneous recovery or maturation even without intervention [3]. In line with this, it may be difficult to separate the effects of intervention as opposed to natural development at such an early age [3,40]. One way to estimate change above natural development could be to contrast individual development against GMFM-66 reference percentiles for children at separate GMFCS levels [34]. Based on this procedure, our results suggest that children later classified at GMFCS levels I-III possibly improved their gross motor abilities more than expected during the Small Step intervention program but not necessarily more during the training periods targeting mobility. However, these results must be interpreted with caution since the reference curves are based on data containing large individual variability [33,34,35]. Larger intervention studies are required to investigate this further.

### 4.3. Strengths and Limitations

The single-subject research design enabled the exploration of training effects for infants at risk of the most severe CP types. This may be considered a strength, given the limited research on early interventions for non-ambulant children with CP and GMFCS levels IV–V. Another strength was the investigation of intervention fidelity indicating that the therapists implemented the Small Step program according to the general intervention principles and coaching strategies. This may contribute to explaining why there was little difference in treatment effect between the various intervention periods. The parents may have integrated the training principles and continued with the training after the specific training period was completed.

The first and foremost limitation was that we did not have enough repeated measures during the intervention periods to preclude effects caused by tiredness, pain, or other factors. Optimally, we should have had a minimum of three testing points during each training step [13]. Furthermore, the HAI is still not validated for infants with a high risk of bilateral CP; thus, raw scores were used in the analysis. The HAI interval level data may have yielded results more representative of true changes. Lastly, blinded scoring was only performed for the HAI. Thus, it cannot be ruled out whether this affected the results although the therapists responsible for the testing were not involved in the provision of the intervention.

## 5. Conclusions

Our study indicates that the Small Step program facilitated motor development in children at high risk of CP, with no specificity of training. Children who responded best had moderate HINE global scores (28–50) at three months of corrected age. In contrast, children with low HINE global scores (≤25) and severely delayed motor function showed only minor improvements and were classified at GMFCS and MACS levels IV and V. Further research is required to investigate the most effective intervention approaches, especially for infants at high risk of severe CP.

## Figures and Tables

**Table 1:** Demographic characteristics of the included children.

Participants (*n*)	12
Gender: Female/Male (*n*)	4/8
Gestational age	
Mean age: weeks (min–max; ±SD)	35.3 (24.6–41.4; ±5.2)
Extreme preterm/preterm/term birth/SGA (*n*)	1/4/6/1
Diagnosis at two years of age	
Bilateral spastic CP	4
Dyskinetic CP	4
Unspecified CP	1
Normal/delayed motor development	1/2
Additional impairments	
Epilepsy	2
Visual impairment	2
Hearing impairment	2
Hydrocephalus, shunt	2
Gastrostomy	3
High comorbidity ≥ 3 additional diagnoses	2
Family (*n*)	
Living with both parents: Yes/No	10/2
Mother’s educational levels ^a^ 3/4/5/6/7/8	1/3/0/3/1/2
Father’s educational levels ^a^ 3/4/5/6/7/8	0/3/0/3/3/0
Mother’s mean age at child’s birth: years (min–max; ±SD) ^b^	32 (25–42; ±6)
Father’s mean age at child’s birth: years (min–max; ±SD) ^b^	36 (28–57; ±10)
Number of siblings: 0/1/2/3	2/8/1/1

*n* = number, min = minimum, max = maximum, SD = standard deviation, SGA = small for gestational age, CP = cerebral palsy; ^a^ = According to the eight levels of the national framework of professional certifications, of which eight is the highest level of education, ^b^ = 2 missing.

**Table 2:** Intervention-related data presented for each child.

	Age * in Months (A3)	Randomized to Hand Function (B) or Mobility (C)	Length of Baseline (A1–3), Weeks	Training Periods Completed	Weeks Involved in Intervention	Length of Post-Intervention (A4–5), Weeks
ID1	7.5	B	5	5	35	8
ID2	4.5	B	5	2	21	-
ID3	6.0	C	11	2	22	-
ID4	5.5	C	6	4	35	-
ID5	4.5	C	4	5	43	4
ID6	5.0	B	6	5	34	4
ID7	5.0	C	1	3	18	-
ID8	5.0	C	5	5	43	8
ID9	5.0	C	5	5	43	4
ID10	5.0	B	5	5	47	4
ID11	5.5	B	5	5	40	4
ID12	4.5	C	4	5	37	4

ID = identification number, * = corrected age, A1–3 = first, second, and third data collections at baseline, A4–5 = data collections post-intervention.

**Table 3:** Grouping of participants based on initial Hammersmith Infant Neurological Examination (HINE) global scores at three months of corrected age (3mCA): HINE scores < 25 (Group 1); HINE scores between 25 and 40 (Group 2); and HINE scores >40 (Group 3).

	HINE (3mCA)	MRICS (>3mCA)	GMA (3mCA)	AIMS (3mCA)	PDMS2 FMC *	PDMS2 GMC *	CP (2yCA)	Mini-MACS (2yCA)	GMFCS (2yCA)
Group 1									
ID3	22.0	B	F−	5	6	4	Y	5	5
ID6	24.5	B	F+/−	6	10	10	Y	5	5
ID8	17.5	D	F−	6	15	6	Y	4	5
Group 2									
ID4	34.5	C	F−	7	48	31	Y	2	3
ID5	29.5	B	F−	7	49	13	Y	2	3
ID7	28.0	B	F−	15	36	29	Y	1	2
ID9	29.5	B	F−	9	37	20	Y	3	4
ID12	35.0	C	F+/−	9	38	28	Y	1	1
Group 3									
ID1	50.0	C	F+/−	18	55	43	N	-	-
ID2	42.0	A	F−	6	6	9	Y	5	5
ID10	45.5	D	F+/−	13	62	82	N	-	-
ID11	43.5	B	F+/−	8	64	36	N	-	-

MRICS = Magnetic Resonance Imaging classification system; GMA = General Movements Assessment; AIMS = Alberta Infant Motor Scale; PDMS-2 = Peabody Developmental Motor Scales 2nd edition; FMC = Fine Motor Composite; GMC = Gross Motor Composite * = total change derived during the Small Step intervention; CP = cerebral palsy; Mini-MACS = Mini-Manual Ability Classification System; GMFCS = Gross Motor Function Classification System; 2yCA = two years of corrected age; ID = identification number; A = maldevelopments; B = predominant white matter injury; C = predominant grey matter injury; D = miscellaneous; F− = absent fidgety movements; F+/− = absent or sporadic fidgety movements; Y = Yes; N = No.

## Data Availability

The data presented in this study are available on request from the corresponding author due to ethical reasons.

## Supplementary Materials

The following supporting information can be downloaded at: https://www.mdpi.com/article/10.3390/jcm13175287/s1, Table S1: Fine and gross motor development measured with Peabody Developmental Motor Scales 2nd edition (PDMS-2).
