# Supplementary material for: The Small Step Early Intervention Program for Infants at High Risk of Cerebral Palsy: A Single-Subject Research Design Study
Source: J Clin Med. 2024 Sep 6;13(17):5287. doi: 10.3390/jcm13175287 (PMC11396191; doi:10.3390/jcm13175287)
Supplement: Supplementary file 1 [file jcm-13-05287-s001.zip › jcm-3155952-supplementary.pdf]

Table S1: Fine and gross motor development measured with Peabody Developmental Motor Scales 2nd edition (PDMS-2). The fine and gross motor composite raw scores (FMC and GMC, respectively) from timepoint to timepoint at baseline (A1-A3), after each intervention foci (B=Hand function, C=Mobility, D=Communication), and post intervention (A4-A5). The numbers in bold denotes FMC and GMC raw score at the first baseline testing, at the end point of the Small Step program, and at follow-up of two years corrected age (2yCA). The numbers presented after the first baseline testing for each child, and in the consecutive columns including Post intervention, denotes the change in raw scores compared to the previous timepoint. In the column Total change after intervention, the summed change in raw score after the completed intervention periods is given. A positive change of ≥6 raw scores is color-coded green. Changes between -5 and +5 raw scores are marked with blue to indicate no real change, while negative changes from -6 and lower are marked in orange to indicate a decline compared to the preceding timepoint.

	Baseline			*Intervention: Hand: B; Mobility: C; Communication: D*					*Post intervention*		*Total change after intervention*	*End point*	*Follow-up 2yCA*
ID1	*A1*	*A2*	*A3*	*B1*	*C1*	*D*	*B2*	*C2*	*A4*	*A5*	*A3-C2*	*A5*	
FMC	**45**	+5	+10	+17	+14	+10	+5	+9	0	+3	+55	**118**	**135**
GMC	**35**	+6	+1	+16	+10	+11	+3	+3	+2	+10	+43	**97**	**114**
ID2	*A1*	*A2*	*A3*	*B1*	*C1*	*D*	*B2*	*C2*	*A4*	*A5*	*A3-C1*	*B1*	
FMC	**19**	+2	+6	+4	+2	-	-	-	-	-	+6	**33**	-
GMC	**17**	+1	+5	+8	+1	-	-	-	-	-	+9	**32**	-
ID3	*A1*	*A2*	*A3*	*C1*	*B1*	*D*	*C2*	*B2*	*A4*	*A5*	*A3-B1*	*B1*	
FMC	**8**	+7	+6	+3	+3	-	-	-	-	-	+6	**27**	**28**
GMC	**6**	0	+8	+3	+1	-	-	-	-	-	+4	**18**	**10**
ID4	*A1*	*A2*	*A3*	*C1*	*B1*	*D*	*C2*	*B2*	*A4*	*A5*	*A3-C2*	*B2*	
FMC	**24**	+3	+15	+10	+10	+13	+15	-	-	-	+48	**90**	**134**
GMC	**18**	+11	+11	+2	+6	+9	+14	-	-	-	+31	**71**	**90**
ID5	*A1*	*A2*	*A3*	*C1*	*B1*	*D*	*C2*	*B2*	*A4*	*A5*	*A3-B2*	*A5*	
FMC	-	**10**	+19	+14	+9	+18	+7	+1	+9	+6	+49	**93**	**116**
GMC	-	**24**	+7	+2	+7	+1	0	+3	+5	+3	+13	**52**	**75**
ID6	*A1*	*A2*	*A3*	*B1*	*C1*	*D*	*B2*	*C2*	*A4*	*A5*	*A3-C2*	*A5*	
FMC	**22**	+1	+3	+5	+5	0	0	0	+1	+1	+10	**37**	**34**
GMC	**15**	+5	+4	+8	+3	0	-1	0	-2	-4	+10	**28**	**30**

ID7	*A1*	*A2*	*A3*	*C1*	*B1*	*D*	*C2*	*B2*	*A4*	*A5*	*A3-D*	*D*	
FMC	-	-	**49**	+2	+26	+8	-	-	-	-	+36	**85**	**135**
GMC	-	-	**38**	+6	+20	+3	-	-	-	-	+29	**67**	**114**
ID8	*A1*	*A2*	*A3*	*C1*	*B1*	*D*	*C2*	*B2*	*A4*	*A5*	*A3-B2*	*A5*	
FMC	**19**	+11	+5	+2	+3	+6	-1	+5	+2	+3	+15	**55**	**58**
GMC	**22**	0	+6	-2	+1	+3	+3	+1	-2	-1	+6	**31**	**39**
ID9	*A1*	*A2*	*A3*	*C1*	*B1*	*D*	*C2*	*B2*	*A4*	*A5*	*A3-B2*	*A5*	
FMC	**21**	+9	+3	+8	+8	+19	0	+2	+1	+5	+37	**76**	**93**
GMC	**26**	+1	+1	+2	+3	+2	+3	+10	0	+1	+20	**49**	**56**
ID10	*A1*	*A2*	*A3*	*B1*	*C1*	*D*	*B2*	*C2*	*A4*	*A5*	*A3-C2*	*A5*	
FMC	**41**	+2	+6	+29	+7	+6	+13	+7	0	+4	+62	**115**	**124**
GMC	**37**	+3	0	+19	+30	+5	+18	+10	+1	+1	+82	**124**	**141**
ID11	*A1*	*A2*	*A3*	*B1*	*C1*	*D*	*B2*	*C2*	*A4*	*A5*	*A3-C2*	*A5*	
FMC	**41**	+4	+3	+19	+2	+14	+14	+15	+4	+2	+64	**118**	**135**
GMC	**24**	+7	+4	+15	+6	+5	+8	+2	+10	+3	+36	**84**	**118**
ID12	*A1*	*A2*	*A3*	*C1*	*B1*	*D*	*C2*	*B2*	*A4*	*A5*	*A3-B2*	*A4*	
FMC	**14**	+14	+4	+10	+18	+6	+3	+1	0	-	+38	**70**	**106**
GMC	**28**	+2	0	+3	+3	+12	+5	+5	+1	-	+28	**59**	**106**
